# Distribution and Variation of Serotypes and Pneumococcal Surface Protein A Clades of *Streptococcus pneumoniae* Strains Isolated From Adult Patients With Invasive Pneumococcal Disease in Japan

**DOI:** 10.3389/fcimb.2021.617573

**Published:** 2021-03-19

**Authors:** Bin Chang, Yuki Kinjo, Masatomo Morita, Kosuke Tamura, Hiroshi Watanabe, Yoshinari Tanabe, Koji Kuronuma, Jiro Fujita, Kengo Oshima, Takaya Maruyama, Shuichi Abe, Kei Kasahara, Junichiro Nishi, Tetsuya Kubota, Makoto Ohnishi, Shigeru Suga, Kazunori Oishi

**Affiliations:** ^1^Department of Bacteriology I, National Institute of Infectious Diseases, Tokyo, Japan; ^2^Department of Bacteriology, The Jikei University School of Medicine, Tokyo, Japan; ^3^Jikei Center for Biofilm Science and Technology, The Jikei University School of Medicine, Tokyo, Japan; ^4^Department of Intelligent Network for Infection Control, Tohoku University Graduate School of Medicine, Sendai, Japan; ^5^Toyama Institute of Health, Toyama, Japan; ^6^Department of Infection Control and Prevention, Kurume University School of Medicine, Fukuoka, Japan; ^7^Department of Respiratory Medicine, Niigata Prefectural Shibata Hospital, Niigata, Japan; ^8^Department of Respiratory Medicine and Allergology, Sapporo Medical University School of Medicine, Hokkaido, Japan; ^9^Department of Infectious, Respiratory, and Digestive Medicine, Faculty of Medicine, University of the Ryukyus, Okinawa, Japan; ^10^Department of Infection Control and Laboratory Diagnostics, Internal Medicine, Tohoku University Graduate School of Medicine, Miyagi, Japan; ^11^Department of Medicine, National Hospital Organization, Mie Hospital, Mie, Japan; ^12^Yamagata Prefectural Central Hospital, Yamagata, Japan; ^13^Center for Infectious Diseases, Nara Medical University, Nara, Japan; ^14^Department of Microbiology, Kagoshima University Graduate School of Medical and Dental Sciences, Kagoshima, Japan; ^15^Department of Respiratory Medicine and Allergology, Kochi Medical School, Kochi University, Kochi, Japan; ^16^National Hospital Organization Mie National Hospital, Mie, Japan

**Keywords:** *Streptococcus pneumoniae*, invasive pneumococcal disease, serotype, pneumococcal surface protein A clade, vaccine, Japan, adults

## Abstract

Pneumococcal surface protein A (PspA) is a surface protein of *Streptococcus pneumoniae* that may be a candidate antigen for new pneumococcal vaccines. This study investigates the distribution of PspA clades of the causative strains of adult invasive pneumococcal disease (IPD) in Japan. Of the 1,939 strains isolated from cases of adult IPD during 2014–2019, the PspA clades of 1,932 (99.6%) strains were determined, and no *pspA* was detected in the remaining 7 strains (0.4%). PspA clades 1–6 were detected in 786 (40.5%), 291 (15.0%), 443 (22.8%), 369 (19.0%), 33 (1.7%), and 6 (0.3%) strains, respectively. New PspA clades (0.2%) were identified in two non-typeable and two serotype 35B pneumococci. The proportions of clade 1 and clade 2 showed significantly decreased and increased trends, respectively. Furthermore, the PspA clade of pneumococcal strains was partially serotype- and sequence type-dependent. The majority of strains belonging to serotypes contained in both the 13-valent pneumococcal conjugate vaccine (PCV13) and the 23-valent pneumococcal polysaccharide vaccine (PPSV23) belonged to PspA clades 1 or 3. In contrast, the distribution of clades in non-vaccine serotypes was wider than that of vaccine serotype pneumococci. Our findings demonstrate that almost all pneumococcal strains from adult IPD express PspA clades 1–4, especially for non-vaccine serotypes. These results may be useful for the development of a new pneumococcal vaccine with PspA.

## Introduction

*Streptococcus pneumoniae* is the most common cause of pneumonia, bloodstream infections, and meningitis in young children and adults 65 years or older (Centers for Disease Control and Prevention, 2020). To date, 100 pneumococcal capsular serotypes have been identified (Ganaie et al., 2020). Currently, vaccines for the prevention of *S. pneumoniae* infections include the 23-valent pneumococcal polysaccharide vaccine (PPSV23) and the 13-valent pneumococcal conjugate vaccine (PCV13). These vaccines only cover some pneumococcal serotypes and cannot protect against infections due to non-vaccine serotypes and unencapsulated *S. pneumoniae* (Briles et al., 2019). The 7-valent pneumococcal conjugate vaccine (PCV7) was available for children in Japan in 2010 and was replaced by PCV13 in November 2013. PCVs have been included in the national immunization program (NIP) since April 2013. After the introduction of PCVs, the incidence of IPD in children aged <5 years decreased by >50%; however, this has not decreased further (Suga et al., 2015). PCV13 and PPSV23 have already been licensed for adults, and PPSV23 for those aged ≥65 years has been included in the Japanese NIP from October 2014. We have been studying epidemiology and clinical features of IPD in adults residing in 10 prefectures of Japan (the same area as this study) since 2013 (Fukusumi et al., 2017; Shimbashi et al., 2019; Shimbashi et al., 2020).

The direct and indirect protective effects of PCV have been reported worldwide (Berical et al., 2016). However, an increase in invasive pneumococcal disease (IPD) caused by non-vaccine serotypes (serotype replacement) occurred after the introduction of PCV, especially in children (Hausdorff and Hanage, 2016). The serotype replacement was also observed in adult IPD (Kendall et al., 2016). Because of the limitations of capsular polysaccharide vaccines, there is an urgent need to develop new, effective, and affordable pneumococcal vaccines covering a wide range of serotypes. These candidates include protein-based pneumococcal vaccines using conserved pneumococcal antigens, such as surface-exposed protein, and detoxified pneumolysin.

Pneumococcal surface protein A (PspA) is a choline-binding protein on the cell surface of almost all pneumococcal strains that inhibits the complement-mediated clearance of pneumococci (McDaniel et al., 1984). PspA comprises five domains, namely, a signal peptide, an α-helical highly charged domain, a proline-rich region domain, a choline-binding domain, and a short hydrophobic tail (Hollingshead et al., 2000). The α-helical highly charged domain has an α-helical coiled-coil structure that is further divided into regions A, B, and C (McDaniel et al., 1994). The B region is the clade-defining region, and amino acid residues 192 and 260 of PspA are protection-eliciting epitopes. The sequence variations of this region are used to classify pneumococcal strains into three families and six clades (Pimenta et al., 2006). It was reported that almost 100% of clinical isolates from cases of IPD in adults and non-IPD in children in Japan belonged to either PspA family 1 (clades 1 and 2) or 2 (clades 3–5) (Piao et al., 2014; Kawaguchiya et al., 2018). However, the *S. pneumoniae* strains analyzed in these studies were from adult cases of IPD before the introduction of PCVs or from noninvasive diseases in children. Therefore, the PspA clade distribution of a relatively large number of *S. pneumoniae* strains from adult IPD cases after PCV introduction has not yet been reported.

This study determined the PspA clade of 1,932 *S. pneumoniae* strains isolated from adult IPD cases in Japan between 2014 and 2019 to elucidate whether PCV introduction influenced the PspA clade distribution. We also analyzed the relationships between PspA clade, serotype, and clonal complex (CC).

## Materials and Methods

### IPD Case Definition and Bacterial Strains

The adult IPD Study Group implemented population-based surveillance in Japan in 2013 of IPD cases occurring in people over the age of 15 years old who resided in 10 prefectures of Japan (Hokkaido, Miyagi, Yamagata, Niigata, Mie, Nara, Kochi, Fukuoka, Kagoshima, and Okinawa). When IPD occurred, the clinical information and the causative pneumococcal strains were simultaneously collected and sent to the National Institute of Infectious Diseases (NIID). The clinical information included the patient’s sex, age, and history of PCV13 and PPSV23 vaccinations.

A case of IPD was defined as the detection of pneumococci by bacterial culture from normally sterile sites. Our study analyzed *S. pneumoniae* strains isolated from adult IPD patients from January 2014 to December 2019. One isolate per case was included.

### Serotyping, Multilocus Sequencing Typing (MLST), and PspA Clades

All *S. pneumoniae* strains were plated on Columbia agar with 5% sheep blood (Nippon Becton Dickinson Co. Ltd., Tokyo, Japan). The quellung reaction was used for serotype determination with pneumococcal antisera (Statens Serum Institut, Copenhagen, Denmark). Serotype 11A/E and non-typeable (NT) were determined as described previously (Shimbashi et al., 2020).

The genomic DNA of the pneumococcal isolates was purified using a High Pure PCR Template Purification Kit (Roche Diagnostics, Tokyo, Japan). MLST was performed as described by Enright and Spratt (1998). Allelic numbers and sequence types (STs) were assigned using the pneumococcal MLST website (https://pubmlst.org/spneumoniae/). Strains where ≥5/7 alleles were identical were classified as a CC (Gertz et al., 2003). PspA clade determination was performed as described by Pimenta et al. (2006). For strains where the PCR fragment was not amplified, an additional PCR reaction was performed using primers upstream (*pspA*-up: 5′-CACACGAGATTATGCTAGTC-3′) and downstream (*pspA*-dn: 5′-CTGCTCCTTGAGCAAAAGAG-3′) of *pspA*, and if a PCR fragment and *pspA* sequence was then obtained, the PspA clade was determined. For strains having sequences that were <90% identical to the sequences of known clades 1–6, BLAST search in nucleotide database of NCBI was performed.

### Whole Genome Sequencing Analysis

Genomic DNA libraries of the pneumococcal strains in which *pspA* could not be detected by PCR were constructed using the Nextera XT DNA sample prep kit (Illumina, San Diego, CA, USA) and then sequenced using an MiSeq (Illumina). After genome assembly was performed using the SPAdes version 3.13.1 with the careful option and a read coverage cutoff value of 10 (Bankevich et al., 2012), homology search of *pspA* gene on the genomes was performed by GENETYX-MAC (GENETYX, Tokyo, Japan).

The whole genome sequences (accession numbers: DRX251224-DRR251230) as well as new (LC597020-LC597021) and deletion mutant (LC597022-LC597023) *pspA* sequences of pneumococci have been deposited in the DNA Data Bank of Japan.

### Statistical Analysis

The proportions of each clade were compared using *χ^2^*-test or Fisher’s exact test. The Mantel–Haenszel test was used to reveal the trend of the proportions for each clade from 2014 to 2019. Multiple comparisons were corrected using Bonferroni’s method. *P* values <0.05 were considered to be significant. All statistical analyses were performed using IBM SPSS Statistics version 24 (IBM Corp., Armonk, NY, USA).

### Ethics Statement

This study was reviewed and approved by the Ethics Committee of the NIID and was conducted according to the principles expressed in the Declaration of Helsinki. Informed consent was waived because the data did not contain any patient identifiers, and the samples were taken in the course of standard patient care.

## Results

### Characteristics of IPD Cases

A total of 1,963 IPD cases occurred from January 2014 to December 2019 that were reported to the Adult IPD Study Group. Of them, 24 cases were excluded from our study because the *S. pneumoniae* strains isolated from 20 cases had died, and live bacteria could not be isolated from the remaining 4 cases. The *S. pneumoniae* strains from the remaining 1,939 IPD cases underwent serotyping, MLST, and PspA clade determination. Another 5 cases were excluded because of incomplete clinical data. The remaining 1,934 patients were 15–103 years old, with a median age of 71. Sixty percent of the patients were men.

### Serotype Distribution of the Pneumococcal Strains

The most prevalent serotypes were 3 (230 strains; 11.9%), 12F (195 strains; 10.1%), 19A (166 strains; 8.6%), 10A (148 strains; 7.7%), and 23A (135 strains; 7.0%). In total, 32.5% of them belonged to PCV13 serotypes and 63.0% belonged to PPSV23 serotypes.

The prevalence of each pneumococcal serotype for each year is shown in the **Supplementary Figure 1**. The number of IPD cases each year was 203, 220, 291, 416, 391, and 418 between 2014 and 2019. The isolation rates of serotypes 3 and 19A peaked in 2015 and decreased from 2016. Conversely, the isolation rate of 12F increased from 2016, peaked in 2017, and decreased in 2018 (Shimbashi et al., 2019) before further decreasing in 2019 (**Supplementary Figure 1**). During 2014–2019, the isolation rates of pneumococcal serotypes 10A and 23A were consistently high, whereas those of the PCV7 serotypes were low. The coverage rate of the pneumococcal strains by PCV13 decreased from 44.8% to 27.0%. On the other hand, the coverage rate of PPSV23 remained >60% from 2014 to 2018, decreasing by approximately 5% in 2019.

### PspA Clades of the Pneumococcal Strains

We determined the *pspA* sequences of 1,932/1,939 (99.6%) *S. pneumoniae* strains. The sequences of 1,928 strains (99.4%) shared >90% identity with the sequences of clades 1–6. Two serotype 35B CC558 strains, which were isolated in 2018, had deletions between 601 and 948 bp and between 601 and 957 bp of *pspA*, respectively. Deletion of amino acid residues was noted at positions 201-316 and 201-319, but no stop codons were found. The sequences upstream and downstream of the deleted region of the two strains were identical to each other and also showed high homology with the sequence of clade 4. No similar deletion sequence could be found in the NCBI database. Because PspA between amino acid residues 192 and 260 has been reported to have protection-eliciting epitopes (McDaniel et al., 1994), it is possible that the two strains have different antigenicity with clade 4. Therefore, the *pspA* genes of the two strains were determined to be a new clade (**Table 1**).

**Table 1:** Number of the pneumococcal strains with different serotypes and different clonal complexes (CCs).

Serotype	CC^#^	Clade 1	Clade 2	Clade 3	Clade 4	Clade 5	Clade 6	New clade	Without *pspA* sequence
4*	CC695	0	0	3	0	0	0	0	0
	CC5872	0	0	0	1	0	0	0	0
6B*	CC90	21	1	3	0	0	0	0	0
	CC902	8	0	0	2	0	0	0	1
	CC2224	0	0	3	0	0	0	0	0
	CC338	3	0	0	0	0	0	0	0
9V*	CC166	0	0	2	2	0	0	0	0
	CC280	0	1	4	0	0	0	0	0
14*	CC13	18	0	0	0	0	0	0	0
	CC343	3	0	0	0	0	0	0	0
18C*	CC870	0	0	0	2	0	0	0	0
	CC3594	3	0	0	0	0	0	0	0
19F*	CC236	0	0	34	2	2	0	0	2
	CC242	0	0	0	0	0	0	0	2
23F*	CC242	0	0	0	0	9	0	0	0
	CC1437	0	0	0	0	9	0	0	0
	CC338	0	2	0	0	2	0	0	0
1*	CC306	3	0	0	0	0	0	0	0
3*	CC180	206	3	8	0	1	0	0	0
6A*	CC3113	11	0	0	0	0	0	0	0
	CC81	0	0	7	0	0	0	0	0
	CC90	2	0	0	0	0	0	0	0
7F*	CC191	0	0	36	0	0	0	0	0
19A*	CC320	0	0	6	0	0	0	0	0
	CC2331	0	0	34	0	0	0	0	0
	CC3111	0	0	124	2	0	0	0	0
10A^§^	CC1263	40	0	0	0	0	0	0	0
	CC5236	104	0	0	1	0	0	0	0
15B^§^	CC199	0	0	0	30	0	0	0	0
22F^§^	CC433	105	0	0	0	0	0	0	0
33F^§^	CC717	21	0	0	0	0	0	0	0
9N^§^	CC66	0	0	2	0	0	0	0	0
12F^§^	CC4846	0	0	117	0	0	0	0	0
	CC6945	0	77	0	0	0	0	0	0
8^§^	CC2234	0	0	0	0	1	0	0	0
	CC3500	2	0	0	0	0	0	0	0
11A/E^§^	CC99	0	1	0	71	0	0	0	0
20^§^	CC4745	20	0	0	0	0	0	0	0
15A	CC63	0	0	0	108	0	0	0	1
	CC81	0	0	1	0	0	0	0	1
15C	CC199	0	0	0	21	0	0	0	0
23A	CC338	76	39	0	0	0	0	0	0
24F	CC2572	0	36	0	0	0	0	0	0
24B	CC2572	0	0	3	0	0	0	0	0
	CC2754	0	0	8	0	0	0	0	0
6C	CC2924	1	40	0	0	0	0	0	0
	CC2923	0	0	0	11	0	0	0	0
	CC5241	0	31	0	1	0	0	0	0
7C	CC2758	12	0	0	0	0	0	0	0
38	CC6429	26	0	0	0	0	0	0	0
35B	CC156	0	0	8	0	0	0	0	0
	CC2755	0	0	0	42	0	0	0	0
	CC558	0	1	2	53	0	0	2	0
34	CC3116	29	0	0	0	0	0	0	0
	CC7338	14	0	0	0	0	0	0	0
31	CC11184	0	26	0	0	0	0	0	0
37	CC447	0	0	0	0	0	6	0	0
Non-typeable	CC230	0	0	0	0	0	0	1	0
	CC338	0	1	0	0	0	0	0	0
	CC2331	0	0	1	0	0	0	0	0
	CC2572	0	1	0	0	0	0	0	0
	CC3116	1	0	0	0	0	0	0	0
	CC15490	0	0	0	0	0	0	1	0

*Serotypes belonging to PCV13.

^§^Serotypes belonging to PPSV23 but not to PCV13.

^#^Strains where ≥5 of the 7 alleles in MLST analysis are classified as a CC.

Additionally, the sequences of two non-typeable strains, which were isolated in 2019, showed less than 90% identity with all sequences of clades 1–6. The sequence of the CC230 strain between primers SKH2 and LSM12 had 76% identity with that of clade 6, and the CC15490 strain was 72% of that of clade 3. The sequences of the two strains also showed low identity (56%) with each other. BLAST in the NCBI database showed that the sequence of the CC230 strain was 99% identical to that of isolate 34YLE (LT669627) and the sequence of CC15490 strain was 99% identical to that of isolate R34-3088 (LT669632) (Croucher et al., 2017). Therefore, the *pspA* genes of the two strains were determined to be new clades (**Table 1**).

Besides these strains, the *pspA* fragment of seven strains (0.4%) was not amplified by PCR using the two primer sets. To identify the presence or absence of *pspA*, whole genome sequences of these strains were determined and no gene similar to *pspA* was found by homology search. Therefore, *pspA* is absent in the seven strains. All seven strains were isolated in 2019, and their serotypes were 19F, 15A, and 6B in four, two, and one strain, respectively (**Table 1**).

In summary, 786 (40.5%), 291 (15.0%), 443 (22.8%), 369 (19.0%), 33 (1.7%), and 6 (0.3%) strains belonged to clades 1–6, respectively. Four (0.2%) strains possessed new clades. The *pspA* gene was not detected in seven (0.4%) strains.

**Figure 1:**
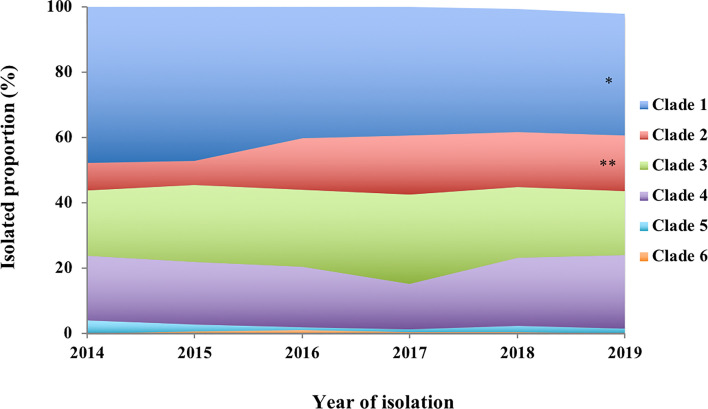
Annual proportion of PspA clades 1–6 among the *S. pneumoniae* strains isolated from invasive pneumococcal diseases in 2014−2019. Mantel–Haenszel test of trends corrected using Bonferroni’s method to perform an analysis of the trends for the proportions of each clade from 2014 to 2019. *p < 0.05, **p < 0.01.

The annual distribution of PspA clades of the *S. pneumoniae* strains is summarized in **Figure 1**.

The statistical analysis of the trends for the proportions of each clade from 2014 to 2019 revealed significantly decreased and increased trends for the proportions of clade 1 and clade 2, respectively (**Figure 1**). Clades 5 and 6 had lower isolation rates than the other clades during the study period (<5%). The total isolation rates of PspA clades 1–4 were 96.1%, 97.3%, 98.3%, 98.8%, 97.2%, and 96.4% from 2014 to 2019, respectively.

### Relationship Between PspA Clade and Serotype and Between PspA Clade and Clonal Complexes

**Figure 2:**
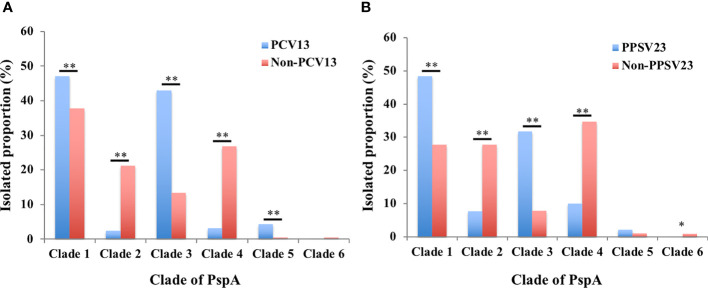
Isolation proportions of PspA clades 1–6 among the *S. pneumoniae* strains of vaccine and non-vaccine serotypes. **(A)** PCV13 and non-PCV13 serotypes, **(B)** PPSV23 and non-PPSV23 serotypes. The proportions of vaccine and non-vaccine serotypes were compared using *χ^2^*-test (clades 1–5) or Fisher’s exact test (clade 6) corrected using Bonferroni’s method. ^*^ *p* < 0.05, ^**^ *p* < 0.01. PCV13: 13-valent pneumococcal conjugate vaccine; PPSV23: 23-valent pneumococcal polysaccharide vaccine.

We compared differences in the proportions of PspA clades of the *S. pneumoniae* strains belonging to the vaccine and non-vaccine serotypes. The *S. pneumoniae* strains belonging to PCV13 and PPSV23 had higher rates in PspA clades 1 and 3. On the other hand, the non-PCV13 and non-PPSV23 strains had higher rates in PspA clades 2 and 4 (**Figure 2**).

The PspA clades of the *S. pneumoniae* strains that belonged to the vaccine serotypes and other major serotypes are summarized in **Table 1**, and the major CCs of these serotypes are also recorded. All strains of serotype 14, 7F, 15B, 22F, 33F, 9N, 20, 15C, 16F, 21, 24F, 24B, 7C, 38, 34, 29, 31, and 37 comprised a single clade. The PspA clade for serotypes 18C, 6A, and 12F was CC-dependent. Serotypes 6B, 19F, 23F, 3, 8, 11A/E, 15A, 23A, 23B, 6C, 6D, 35B, and non-typeable strains were found in multiple clades.

The CCs of the seven strains that did not possess *pspA* were CC236 (19F, two strains), CC242 (19F, two strains), CC63 (15A, one strain), CC81 (15A, one strain), and CC902 (6B, one strain). Among the four strains that showed new PspA clades, the two 35B strains were CC558 and the two non-typeable strains were CC230 and CC15490 (**Table 1**).

## Discussion

Our study summarized the distribution of the PspA clades of 1,928 *S. pneumoniae* strains isolated from cases of adult IPD in Japan during 2014–2019. This is the first report of PspA clade distribution of *S. pneumoniae* strains isolated from adult cases of IPD after PCV introduction for children in Japan. The proportions of PspA clades 1–6 were 40.5%, 15.0%, 22.8%, 19.0%, 1.7%, and 0.3%, respectively. A previous study of 68 strains isolated from adult cases of IPD in Japan during 2010–2011 reported that the major PspA clades were 1 (50%) and 3 (28%) (Piao et al., 2014). The other clades 2, 4, and 5 comprised minor proportions of 4%, 9%, and 9% of the cases, respectively. There was no clade 6. The median age of the 68 patients was 68 years, similar to the median age 71 years in this study. Because pneumococcal strains isolated from adult IPD before PCV introduction were limited in Japan, only 68 strains from adult IPD were analyzed in the previous study (Piao et al., 2014). However, we determined PspA clade distribution of 250 pneumococcal strains isolated from pediatric IPD cases that occurred before the introduction of PCVs (Suga et al., 2015), and these data show a trend similar to that obtained from the 68 adult IPD strains. The major PspA clades of the pneumococci from children were 1 (42%) and 3 (32%), whereas the other clades 2, 4, and 5 comprised minor proportions of 4%, 9%, and 13% of the cases, respectively. There was no clade 6 (**Supplementary Figure 2**). These strains were isolated from pediatric IPD patients who resided in 10 prefectures of Japan (Suga et al., 2015); among the 10 prefectures, seven (Hokkaido, Niigata, Mie, Kochi, Fukuoka, Kagoshima, and Okinawa) were the same as those surveyed in this study. Therefore, the PspA clade distribution of 68 strains from adult patients with IPD and 250 strains from pediatric patients with IPD could be used as controls for this study. Because PCVs have been part of the NIP for children in Japan since 2013, our findings suggested that the PspA clade distribution of strains that caused adult IPD changed considerably after PCV introduction in children. During 2014–2019, our data demonstrated that the annual distributions of PspA clade 1 and clade 2 exhibited significantly decreased and increased trends, respectively (**Figure 1**). We compared the PspA clade distribution before and after PCV using pediatric IPD and adult IPD data in 2019, respectively. Compared to the data before PCV, clades 2 and 4 were significantly increased and clades 3 and 5 were significantly decreased after PCV. Although there was a tendency for clade 1 to decrease after PCV, the difference was not statistically significant (**Supplementary Figure 3**). Furthermore, *S. pneumoniae* strains of PCV13 and PPSV23 serotypes had higher rates in PspA clades 1 and 3 (**Figure 2**). These results suggest that the serotype replacement might have influenced (**Supplementary Figure 1**), but only partially, the change in PspA clade distributions.

In this study, 4 strains (0.2%) were classified as new clades because they had the *pspA* sequences that differed from the well-known clades 1–6 (**Table 1**). Moreover, there were 7 *pspA*-negative strains (0.4%). Although the total rate of strains without *pspA* and having new PspA clade is low (0.6%), continuous surveillance would be important to determine if there will be any changes in the distribution of PspA clades.

The protective effects of PCV have been well-recognized worldwide (Berical et al., 2016; Kim et al., 2016). The introduction of PCV7 and PCV13 in children dramatically decreased the incidence of IPD caused by vaccine serotypes, and annual incidence of IPD in children aged <5 years decreased significantly by 57% in 2013 compared with that in 2008 before PCV introduction in Japan (Suga et al., 2015; Nakano et al., 2020). In addition to direct effect, PCV in national immunization programs for children has had a significant indirect effect on pneumococcal diseases in adults (Berical et al., 2016; Kim et al., 2016). Compared with PCV, the protective effects of PPSV23 are controversial. However, a systematic review and meta-analysis reported the effectiveness of PPSV23 against IPD in adults aged >50 years to be 54% (Kraicer-Melamed et al., 2016). PPSV23 was included for those aged ≥65 years in Japanese national vaccine program in 2014. The adjusted vaccine effectiveness of PPSV23 against adult IPD caused by vaccine serotypes was 42.2% (Shimbashi et al., 2020). Furthermore, it was shown that prior vaccination with either PCV13 or PPSV23 decreased the risk of pneumococcal carriage in adults aged ≥65 years (Branche et al., 2018). These studies indicate that not only PCV13 but also PPSV23 have protective effects against pneumococcal diseases. However, because of the serotype replacement, limitations of the PCV13 and PPSV23 exist. Therefore, there is an urgent need for a new, effective, and affordable pneumococcal vaccine that covers a wide range of serotypes for both children and adults in Japan.

The PspA clades of *S. pneumoniae* strains from adult IPD cases were partially serotype- and CC-dependent (**Table 1**). More importantly, our data demonstrated that the isolation rates of the PspA clades 1–4 were maintained at high levels (96.1%–98.8%), and clades 5 and 6 had low rates. The studies of IPD strains from Spain, China, and Korea showed that the rate of PspA clade 5 was 9.1%, 8.2%, and 12.4%, respectively (Rolo et al., 2009; Qian et al., 2012; Yun et al., 2017). The clade 6 was found in only 1 strain from Korea and in no other strains from geographies. However, the number of pneumococci analyzed in these studies was limited as 66, 171, and 190 strains, respectively. Therefore, further studies would be needed to determine the actual rates of clades 5 and 6 among pneumococcal strains from IPD patients in these studies because the rate of clade 5 was 9% in the previous report with 68 adult IPD strains in Japan (Piao et al., 2014). Collectively, our results indicate that new vaccines under development targeting PspA should include at least clades 1–4.

In conclusion, the distribution of the PspA clades of *S. pneumoniae* strains isolated from adult IPD cases during 2014–2019 in Japan were determined. These data may be useful for designing new PspA-based pneumococcal vaccines. We aim to continue examining the serotypes, STs, and PspA clades for an in-depth understanding of *S. pneumoniae* strains that cause IPD in adults.

## Data Availability Statement

The raw data supporting the conclusions of this article will be made available by the authors, without undue reservation.

## Ethics Statement

The studies involving human participants were reviewed and approved by The Ethics Committee of the National Institute of infectious Diseases. Written informed consent from the participants’ legal guardian/next of kin was not required to participate in this study in accordance with the national legislation and the institutional requirements.

## Author Contributions

BC, YK, and KaO designed the study. HW, YT, KoK, JF, KeO, TM, SA, KeK, JN, and TK collected clinical data and bacterial strains. KT performed statistical analyses. BC carried out the microbiological analysis and analyzed the data. MM and MO carried out the whole genome sequencing. BC and SS provided PspA distribution results of pneumococci from children with IPD. BC and YK drafted the manuscript. All authors contributed to the article and approved the submitted version.

## Funding

This work was supported by the Ministry of Health and Labour Sciences HA Program Grant Number JPMH20HA1005, the Japan Society for the Promotion of Science KAKENHI Grant number 19H03705 and the Japan Agency for Medical Research and Development (AMED; JP20fk0108139j1901).

## Conflict of Interest

The authors declare that the research was conducted in the absence of any commercial or financial relationships that could be construed as a potential conflict of interest.
