# Supplementary material for: Distribution and Variation of Serotypes and Pneumococcal Surface Protein A Clades of Streptococcus pneumoniae Strains Isolated From Adult Patients With Invasive Pneumococcal Disease in Japan
Source: Front Cell Infect Microbiol. 2021 Mar 19;11:617573. doi: 10.3389/fcimb.2021.617573 (PMC8044978; doi:10.3389/fcimb.2021.617573)
Supplement: Supplementary file 1 [file DataSheet_1.pdf]

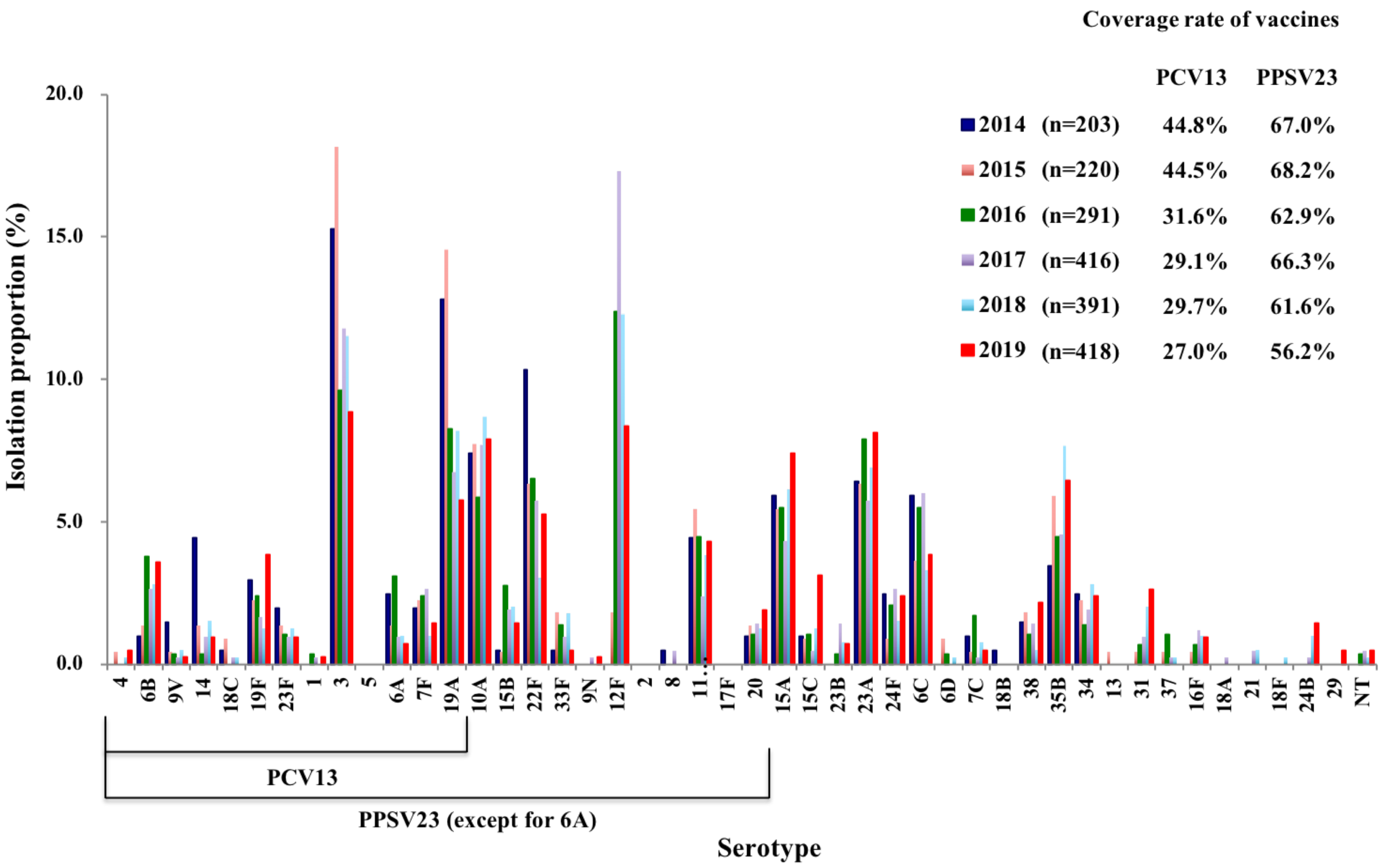

Supplementary Figure 1

Annual serotype distribution and the vaccine coverage of the *S. pneumoniae* strains isolated from invasive pneumococcal disease in adults of Japan between 2014 and 2019. PCV13: 13-valent pneumococcal conjugate vaccine; PPSV23: 23-valent pneumococcal polysaccharide vaccine; NT: serotype non-typeable.

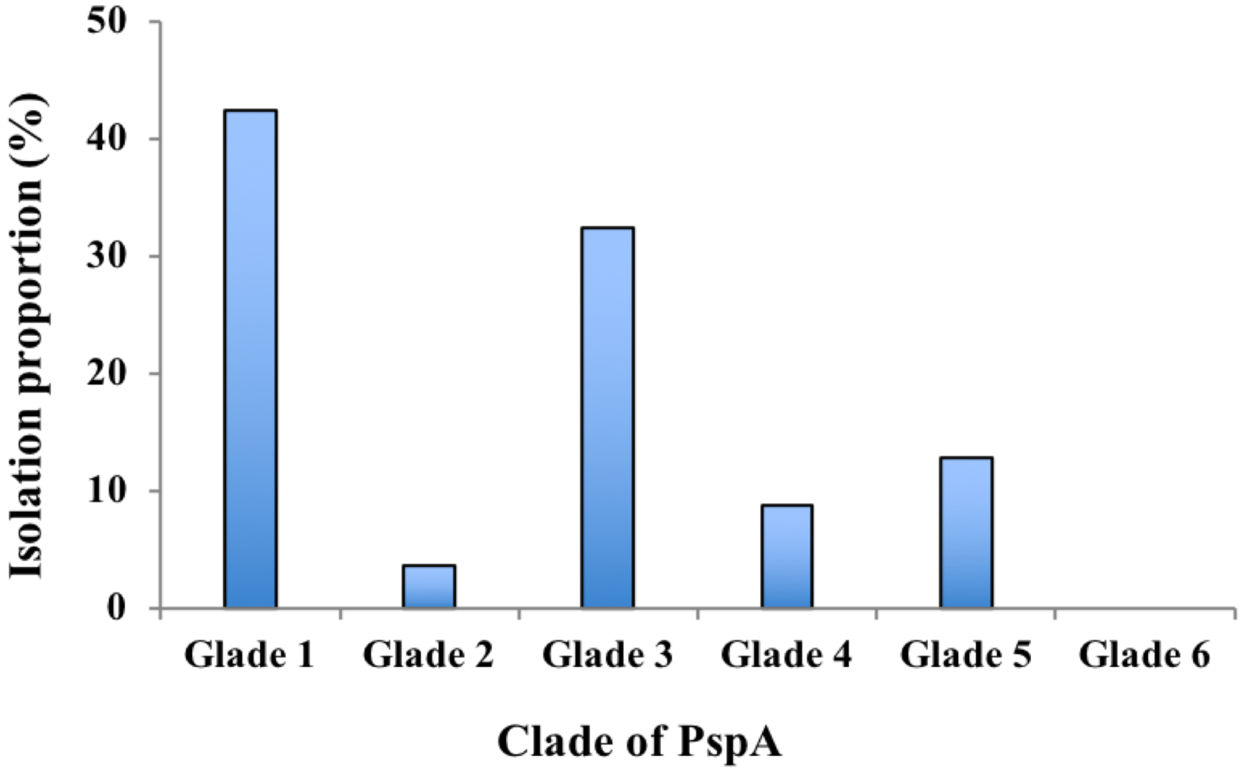

Supplementary Figure 2

Isolation proportions of PspA clades 1–6 among 250 *S. pneumoniae* strains isolated from invasive pneumococcal diseases of children aged <15 years before the introduction of the 7-valent pneumococcal conjugate vaccine in Japan. All the strains were isolated from children under the age of 15 residing in 10 prefectures (Hokkaido, Niigata, Fukushima, Chiba, Mie, Okayama, Kochi, Fukuoka, Kagoshima, and Okinawa) of Japan (Suga S et al., 2015) between 2007 and January 2010 before the introduction of the 7-valent pneumococcal conjugate vaccine in Japan. The IPD case definition was the same as in this study.

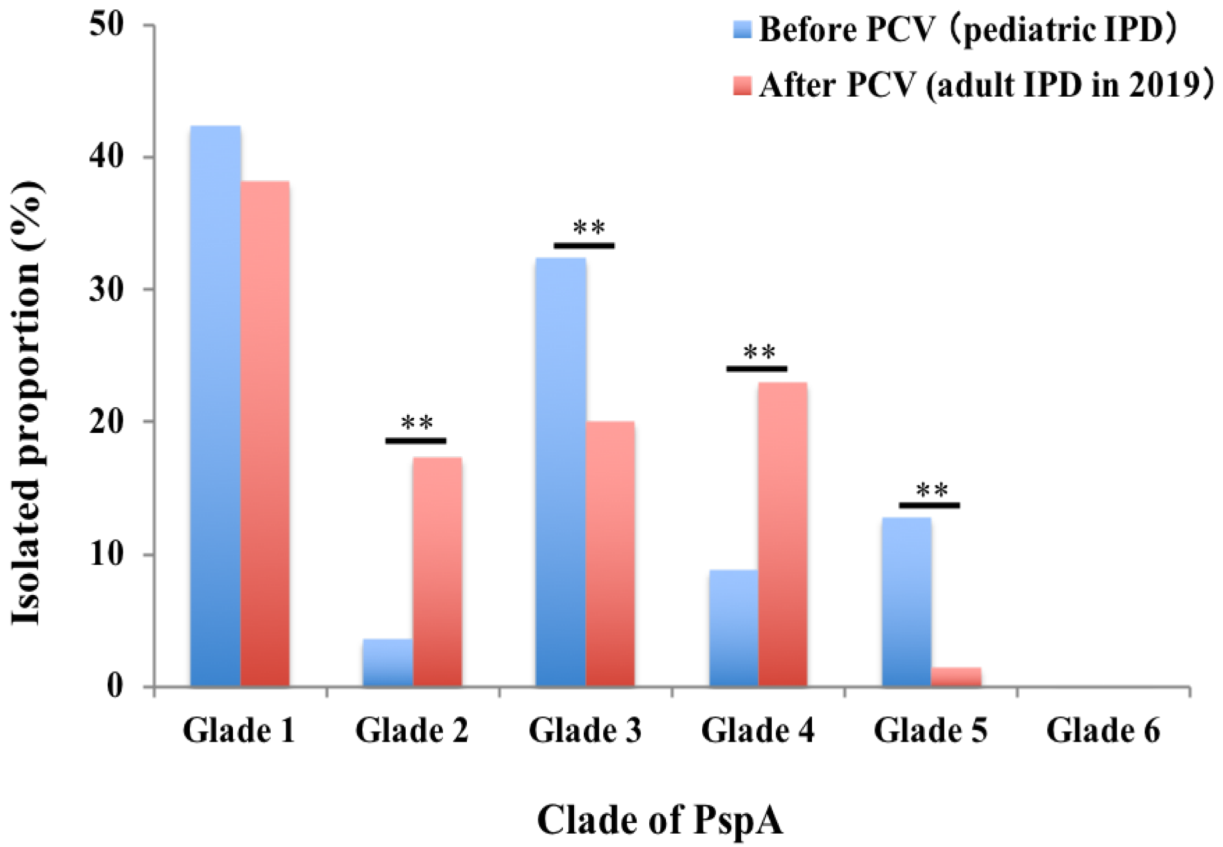

Supplementary Figure 3

Isolation proportions of PspA clades 1–6 among the *S. pneumoniae* strains isolated from pediatric IPD cases before the introduction of PCV7 and strains from adult IPD cases in 2019. The proportions of each clade before and after PCV13 were compared using χ^2-test corrected by Bonferroni's method. ** $p < 0.01$. PCV: pneumococcal conjugate vaccine.
